# Imaging tissue-mimic with light sheet microscopy: A comparative guideline

**DOI:** 10.1038/srep44939

**Published:** 2017-03-21

**Authors:** Jordi Andilla, Raphael Jorand, Omar E. Olarte, Alexandre C. Dufour, Martine Cazales, Yoann L. E. Montagner, Romain Ceolato, Nicolas Riviere, Jean-Christophe Olivo-Marin, Pablo Loza-Alvarez, Corinne Lorenzo

**Affiliations:** 1 ICFO-Institut de Ciences Fotonique, Av. Carl Friedrich Gauss, 3, 08860 Castelldefels, Barcelona, Spain; 2 ITAV, Université de Toulouse, CNRS, UPS, France; 3 Institut Pasteur BioImage Analysis Unit, F-75015, Paris, France; 4 CNRS, UMR-3691, F-75015 Paris, France; 5 Onera, The French Aerospace Lab, F-31100 Toulouse, France

## Abstract

Tissue mimics (TMs) on the scale of several hundred microns provide a beneficial cell culture configuration for *in vitro* engineered tissue and are currently under the spotlight in tissue engineering and regenerative medicine. Due to the cell density and size, TMs are fairly inaccessible to optical observation and imaging within these samples remains challenging. Light Sheet Fluorescence Microscopy (LSFM)- an emerging and attractive technique for 3D optical sectioning of large samples- appears to be a particularly well-suited approach to deal with them. In this work, we compared the effectiveness of different light sheet illumination modalities reported in the literature to improve resolution and/or light exposure for complex 3D samples. In order to provide an acute and fair comparative assessment, we also developed a systematic, computerized benchmarking method. The outcomes of our experiment provide meaningful information for valid comparisons and arises the main differences between the modalities when imaging different types of TMs.

In recent years, tissue mimics (TMs) such as microtissues, spheroids and organoid cultures have become increasingly important in life science research, as they provide a physiologically more relevant environment for cell growth, tissue morphogenesis and stem cell differentiation. In contrast to cell lines cultured in monolayers, these 3D models can be engineered to display most of the hallmarks of native tissue in terms of architecture, cell heterogeneity and self-renewal properties. They thus have great potential for modeling tissue development and disease progression, notably in the context of cancer, heart, and stem cell research, as well as in neurobiology, drug discovery and toxicity testing^1^,^2^.

Fluorescence microscopy is a *sine qua non* tool today to understand cell biology in these TMs. Yet, due to their thick, inhomogeneous and high light-scattering properties, observing TMs at high resolution, in-depth and in real time remains a major technical challenge. Typically, the samples are fixed, serial-sectioned in thin layers, labeled with a given marker and imaged by conventional light microscopy. This strategy, however, does not provide an overall picture of 3D cellular and subcellular processes, and fails to capture dynamic changes over time. Alternatively, 3D imaging techniques such as confocal microscopy can be used to image entire living samples. However, their limited penetration depth and the production of high photo-toxic effects hamper their use for non-invasive, long-term imaging. Over the last decade, Light Sheet Fluorescence Microscopy (LSFM) has emerged as a powerful and versatile solution for long-term live 3D imaging of organism models^3^,^4^,^5^,^6^,^7^,^8^. While most of the literature reports the use of LSFM for imaging developmental biology models, the technique shows great potential in many other application fields^9^, notably for non-invasive TM imaging (Table 1). The first report on LSFM with Multi Cellular Spheroids was illustrated with small (140 μm diameter) spheroids of human pancreatic cells (BxPC3)^10^. This work demonstrated that Selective Plane Illumination Microscopy (SPIM), the most prevalent LSFM, enables imaging such dense models with good spatial resolution, and fully exploits multi-view reconstruction to render a complete 3D vision of the sample. The next step implemented a temperature- and CO_2-_controlled environment to obtain 3D time-lapse images of live cell division dynamics inside large Multi Cellular Tumor Spheroids (MCTS) up to 400 μm-diameter, generated from a Capan-2 colon carcinoma cell line^11^. More recently, the potential of LSFM for imaging human differentiated 3D neural aggregates in fixed as well as in live samples has also been demonstrated^12^.

While SPIM is recognized as a very powerful and promising tool for imaging such samples, it still suffers from various specific limitations. Optical aberrations, absorption and scattering affect both the excitation and the emission of light, resulting in a loss of signal and contrast. Moreover, because of side-illumination, SPIM images are often impaired by stripes and shadows. Overall, these effects severely limit SPIM for imaging deep within complex heterogeneous opaque samples, typically beyond 100 μm.

To address these problems, several image processing tools^13^,^14^,^15^ and hardware^16^,^17^,^18^,^19^,^20^,^21^,^22^ solutions have been proposed and are presently being investigated and evaluated (Table 1). For example, a computational model of stationary noise induced by a pattern affecting an image at random places has been proposed^13^ and successfully applied to remove stripes and shadows on 3D images of MCTS and neurospheres^11^,^12^. From a hardware perspective, Fahrbach and co-workers^23^,^24^ have created a new line-scanned light sheet microscope relying on propagation-invariant “self-reconstructing Bessel beams” which suffer less from light scattering than classic Gaussian-propagating beams. This new technique, however, is impaired by reduced contrast due to the radial extension of the Bessel rings that illuminate out-of-focus regions. This issue has recently been addressed in different ways, either by combining light sheet illumination with a confocal line-detection scheme^25^, by exploiting a non-linear excitation Bessel beam approach^26^,^27^, or by using structured illumination^18^,^28^. More recently, Vettenburg and al. have used another kind of propagation-invariant beam —an Airy beam. By combining Airy beam excitation with a deconvolution algorithm, they have achieved high-contrast imaging over a large field of view. The use of Bessel and Airy beams for 3D TM imaging is also summarized in Table 1.

Given the broad spectrum of LSFM techniques and variants available to date, selecting the optimal LSFM setup for a given biological question is now a major challenge for the research community. Most studies typically describe or compare^29^,^30^,^31^ the use of different LSFM modalities for a specific biological sample or application, using *ad hoc* metrics and codes for qualitative or quantitative validation. Although rigorous in their interpretations, the conclusions obtained in one experimental context may not necessarily apply to another instrument or biological sample. Therefore, the current challenge is to objectively identify the strengths and weaknesses of each method in an unbiased manner. This challenge further emphasizes the need for more reliable and comprehensive community-approved benchmarks^32^.

The present study proposes the first fair comparison of different LSFM techniques in terms of practicality and performance in the specific context of 3D TM imaging. Given the great variety of LSFM systems available, we specifically emphasize the most relevant experimentally validated methods in the hope of providing the community with a comprehensive comparative guideline for 3D TM imaging with light sheet microscopy. Our comparison begins with a description of the experimental setup and the results obtained for each type of light sheet microscopy, and then discusses and summarizes the performance, applicability and limitations of each technique. Although, in this study the comparison is made between different systems and types of TMs, it is not limited to them. The applicability of the methodology can be easily extended to different fluorescence microscopy techniques or other imaging techniques.

## Results

### Tissue mimics: a working definition

**Figure 1:**
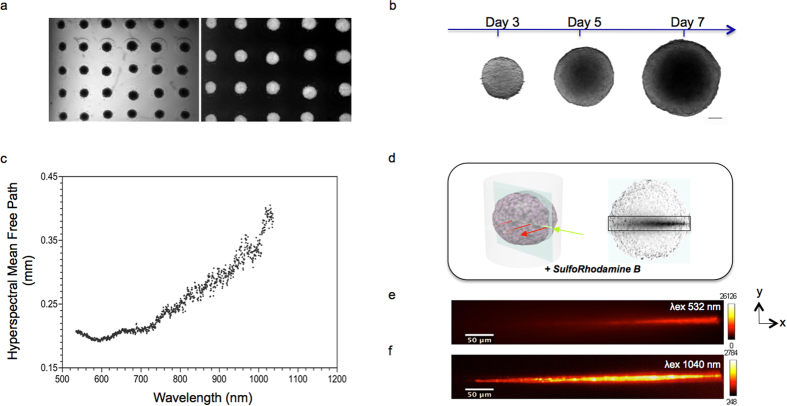
Generation and intrinsic optical properties of TMs. (**a**) Bright field and fluorescence images of MCF10A mammary epithelial MDS production with 3D Petri Dish^®^. MDS were formed at the bottom of agarose micro-wells (**b**) Bright field images of HCT116 colon adenocarcinoma MCTS acquired at different days of culture, scale bar 100 μm (**c**) Mean free path length of photons through MCTS measuring 400 μm in diameter as a function of wavelength. The cross points are the representative experimental data. (**d**) Sketch of the geometry and the intensity profile views of beam at the center, of an MCTS. Background is a white light image. Beams propagate from right to left in the images. (**e–f**) Cross- sectional views (xy) of intensity profiles for (**e**) Gaussian one-photon and (**f**) Gaussian two-photon excitation beams propagating through MCTS measuring 400 μm in diameter and staining with SRB. Color- scale intensity signal has been set to clearly show the propagating beam.

We define TMs as spherical clusters on the scale of several hundred microns (100–500 μm in diameter) of primary or neoplastic/engineered mammalian cells formed by self-assembly. Cells thereby reside in a natural 3D environment and mimic *in vivo* differentiation patterns as well as the 3D network of cell-cell and cell-matrix interactions. We generated four types of TMs in a standardized up-scaled production with controlled parameters (Fig. 1), representing samples with different cell morphology (volume, density, shape, etc.) and cell population heterogeneity. Our TMs thus correspond to samples generally used in different fields of investigation such as cancer biology, cardiac biology and neurobiology: Mammary Duct Spheres (MDS, Fig. 1a) and Multi Cellular Tumor Spheroids (MCTS, Fig. 1b), both generated from established cell lines, and Cardio-Spheroids (CS) and Neurospheres (NS), both generated from cells extracted from mice (cardiomyocytes and central nervous system stem cells, respectively). Currently, there is a large arsenal of user-friendly, inexpensive systems available for up-scaled production of highly reproducible TMs^33^. Apart from NS, which naturally self-organize into a sphere during culture, we produced TMs using either the centrifugation^11^ or the microtissue^®^ 3D Petri Dish^®^ method (Fig. 1a). This allowed us to obtain TMs with a highly reproducible size (coefficient of variation less than 10% in the case of MCTS). We then used different types of fluorescent labeling: TMs expressing a fluorescent nuclear protein (H2B-mCherry) or stained with a fluorescent intercalating agent which binds DNA (Propidium Iodide, PI) and TMs with non-labeled cells in which 0.5 μm fluorescent beads were integrated during culture. For practical reasons and to ensure minimum laboratory rodent wastage, the MCTS were first generated from established colon carcinoma human cancer cell lines (herein HCT116) to characterize their optical properties and develop the evaluation framework.

### Tissue mimics: intrinsic optical properties

Thanks to their inherent radial symmetry, TMs enable us to easily parameterize optical properties as a function of their structure (e.g. aggregation or cellular composition). Light scattering, in particular, is the primary cause of degraded image quality and limited achievable imaging depth^34^. This is because in scattering tissues, the number of ballistic photons that generate the image decay exponentially along the propagation axis. This decay can be characterized by the Mean Free Path (MFP) length of photons^35^. Using an *in vitro*, label-free and hyperspectral optical method optimized for biomedical studies^36^ (Supplementary Note 1, Supplementary Fig. 1 and Supplementary Table 1), we measured the MFP length of fixed, 400 μm-diameter MCTS and found a non-linear relationship between the calculated MFP length and the wavelength of the incident light (Fig. 1c). The measured MFP length ranged from 0.19 mm to 0.40 mm in the visible-near infrared range. Our measurements showed that MFP length increased two-fold from 532 to 1040 nm, thus increasing light propagation for longer wavelengths.

These findings agree with previous reports of spectral dependence for various kinds of tissue and are consistent with a penetration depth measured in the wavelength range of 700–1070 nm (the “optical window”)^37^. Our results were confirmed by fluorescence imaging using a continuous-wave laser with a wavelength of 532 nm (MF ≈ 0 2 mm) and a femtosecond-pulsed near-infrared laser with a wavelength of 1040 nm (MFP ≈ 0 4 mm) propagating through the same MCTS stained with Sulfo-Rhodamine B dye (Fig. 1d–f). We found that the nonlinear excitation was able to propagate across the entire width of the MCTS (Fig. 1f). In contrast, however, when exciting in the linear regime, no light was able to reach beyond the first half of the MCTS (Fig. 1e). The infrared wavelength of the two-photon excitation, having a longer MFP length, propagated inside the MCTS twice as deeply as the visible wavelength in the one-photon excitation. Moreover, we observed that the two-photon beam exhibited almost no scattering behavior (Fig. 1f).

### Experimental setup and evaluation framework

Our comparative analysis was designed in two stages. Firstly, we tested six different light-sheet modalities using a single type of TM. This allowed us to highlight and select the modalities of choice based on resolution, signal-to-noise and contrast measures. Secondly, we further characterized the performance of the selected systems by also varying the type of TM to be observed. In this case, the penetration depth and the properties of the usable imaging volume were measured and quantified.

To ensure the most similar conditions and minimize sample handling in comparing the six different modalities, we used a Single Multimodal LSFM containing all the modalities (Supplementary Fig. 2 and Supplementary Note 2 for a complete description of the setup, and Supplementary Table 2 for a detailed list of components). The system supported both linear (one-photon, hereafter 1 P) and nonlinear (two-photon, hereafter 2 P) excitation regimes, under Gaussian (G) or Bessel (B) beam excitation profiles, thus yielding four possible combinations termed G1P, G2P, B1P & B2P. In all cases, the light sheet was produced by the digitally scanned light sheet microscopy technique or DSLM^38^ (i.e. by rapidly scanning the beam “up and down” during the exposure time). The system also had the option of using a cylindrical lens (termed G1P CL) for accessing the more conventional SPIM modality. Finally, the system included a sCMOS camera in which the scanned illumination beam was synchronized with the “rolling shutter” mode to achieve confocal line detection^25^. The spatial properties of the beams were set to obtain the largest possible field of view (FOV) while maintaining the maximal optical sectioning capability in order to generate high-resolution images (Supplementary Fig. 3, Supplementary Note 2).

We used MCTS stably expressing a nuclear protein (H2B) fused to the mCherry fluorescent protein (Supplementary Fig. 4a), as well as MCTS cultivated with integrated 0.5 μm fluorescent red beads (Supplementary Fig. 4b). All samples were imaged with each of the six different illumination modalities mentioned above (n ≥ 6 for each modality). For the linear modalities, laser power and exposure time were set to standard values used for MCTS imaging^11^. However, these values were empirically adjusted in the non-linear cases in order to obtain data sets with sufficient signal-to-noise ratio (SNR) to make it possible to then analyze the images (Supplementary Table 3).

Quantitative evaluation was carried out using three metrics commonly used in microscopy: SNR (sometimes called signal to background ratio), contrast of the image, and spatial resolution. In the first case the ratio between the maximum of the signal and the background has been used. To find the contrast of the images the Normalized Contrast Index or NCI, has been computed, by finding the magnitude of the differences between adjacent pixels. Finally the resolution of the system has been obtained by determining the 3D PSF in the whole volume of the sample. For more details on the formal definitions and procedures used in this work, please refer to Supp. Note 3.

**Figure 2:**
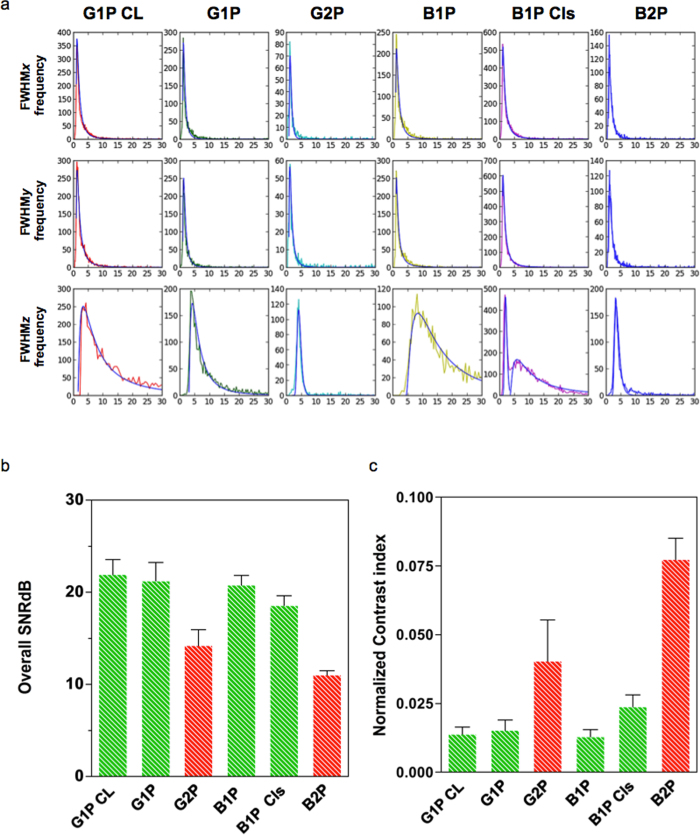
3D PSF, SNR and contrast comparison for MCTS imaged by different light sheet illumination modalities. (**a**) Distributions and lognormal fits (superimposed in blue) of the obtained FHWM values for each modality. Horizontal axes are in μm and vertical axis in counts. (**b**) SNR mean values for each modality in decibels (dB). (**c**) NCI mean values for each modality. All error bars are the s.d.

All measures were automatically extracted from the images using a set of robust and user-friendly image analysis and quantification protocols specifically tailored for the TM models described above. They are available through the open-source Icy platform^39^, providing free and open access to the algorithms and their future iterations (Supplementary Fig. 5, Supplementary Note 3, Supplementary Movie). We first observed that the size of the PSF was similar in both *x* and *y* directions for all modalities (Fig. 2a). For axial resolution, however, we noticed that the full-with at half maximum (FWHM) was twice as large in the B1P case, due to the out-of-plane illumination produced by the side-lobes of the Bessel beam. The fitted distributions obtained for the B1P Cls modality pointed out that the filtering achieved with the rolling shutter was not efficient for all of the beads detected and analyzed. Only a subset of the beads analyzed displayed an axial resolution limited mainly by the thickness of the central lobe of the Bessel beam, while other beads presented a distribution similar to that obtained for the B1P modality (Fig. 2a). In terms of standard deviation, two-photon modalities presented sharper distributions of FWHM than their one-photon counterparts. This was especially true in the *z* direction compared to the G1P CL, B1P and B1P Cls, where there was a large variation of FWHM*z*. This difference reflects the performance of the nonlinear versus the linear regime in terms of reducing scattered and out-of-focus light.

We then plotted the histogram of the overall SNR obtained for each illumination (Fig. 2b), and observed that one-photon modalities (SNR ranging from 17 to 24 dB) were significantly superior to two-photon modalities (SNR ranging from 10 to 17 dB) in terms of fluorescence emission efficiency. These results were expected, given the quadratic dependence of two-photon fluorescence, the laser power and the photon density spread out over a wide FOV. This effect was more pronounced for the B2P modality where the FOV ranged up to twice as large (480 vs. 250 μm). On the other hand, when observing the local contrast measures (given by the NCI), we saw that non-linear modalities (G2P and B2P) yielded the largest values in comparison to any other linear modality (Fig. 2c). Finally, taking both metrics into account (Fig. 2b and c), it was interesting to observe that the G1P and B1P modalities provided similar SNR and NCI, despite the negative impact of the out-of-focus light from the side-lobes of the Bessel beam B1P (Supplementary Fig. 4). Furthermore, confocal line scanning detection on the B1P (BP1 Cls) yielded a higher NCI (0.025 ± 0.004) than the conventional one-photon illumination modalities (ranging from 0.009 to 0.017), while still maintaining similar, although slightly reduced, SNR (18.5 dB ± 1.1).

From this first stage of analysis, we concluded that Bessel modalities with “filtering”, either with confocal line scanning (hard filtering) or two-photon excitation (soft filtering) would generally produce sharper and greater contrast images than those produced with Gaussian modalities in both one-photon and two-photon excitation regimes.

However, it was also evident from our experience that the difficulties of B2P in achieving acceptable SNR to obtain exploitable data sets constituted a real obstacle for obtaining images with this modality. The problem was even worse when working with a FOV as wide as the one used in this first round analysis. Consequently, for a fair comparison, working with a similar level of SNR from one modality to another then was then seen to be mandatory. In addition, the spatial properties of beams should be such that they produce similar FOV with similar optical sectioning capabilities.

**Figure 3:**
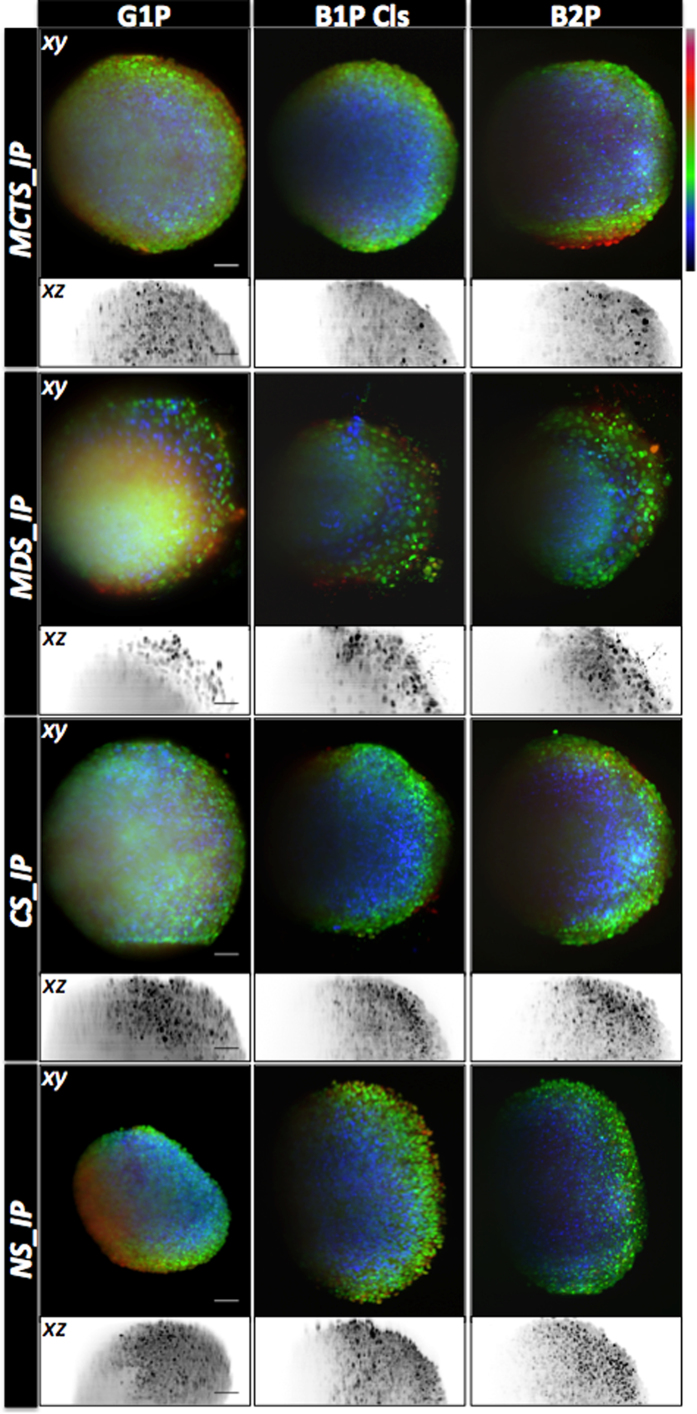
Maximum intensity projection of different TMs imaged by different light sheet modalities. Maximum projections along (x-y) and (x-z) of 3D image stack obtained using the indicated one- side light sheet illumination modality of 400 images (z spacing 1 μm) of MCTS, MDS, CS and NS TMs measuring around 400 μm in diameter and stained with PI are displayed with depth lookup table for (x-y) and inverted gray lookup table for (x-z). For a good visualisation, scale intensity signals were set independently for each of modality. Scale Bar, 50 μm.

Subsequently, we conducted a second set of experiments, where we considered only Bessel modalities with hard and soft filtering capabilities (B1P Cls and B2P). The beams were optimized to obtain fixed FOV of 200 μm and produce good optical sectioning capability, herein 5–6 μm compatible with various TM imaging systems (Supplementary Table 4). The exposure time was fixed at a standard value (500 ms) regardless of the modality. Finally, different TM models, including MCTS, MDS, CS and NS were used to assess the imaging capabilities of the selected light-sheet illumination modalities. All the results were compared with conventional and the most widely used G1P modality. The different TM models imaged here were either stained with PI (Fig. 3) or cultivated with 0.5 μm fluorescent beads. By standardizing the acquisition parameters and sample staining, we ensured that the density of the labeled features and the fluorescence collection capabilities were identical across all systems being compared, and hence that the acquired signal truly reflected the efficiency of fluorescence emission. The remaining parameters needing adjustment were the choice of laser and its power. In addition to the quantitative measures used in the first stage of evaluation, here we developed an additional set of quality metrics specific to LSFM: penetration depth (via two indexes called the Center of Mass or CoM, and the Contrasted Imaging Volume or CIV (see Supplementary Fig. 5, Supplementary Note 3, Supplementary Movie). These measures were obtained using custom image analysis protocols, also available on the Icy platform^39^.

**Figure 4:**
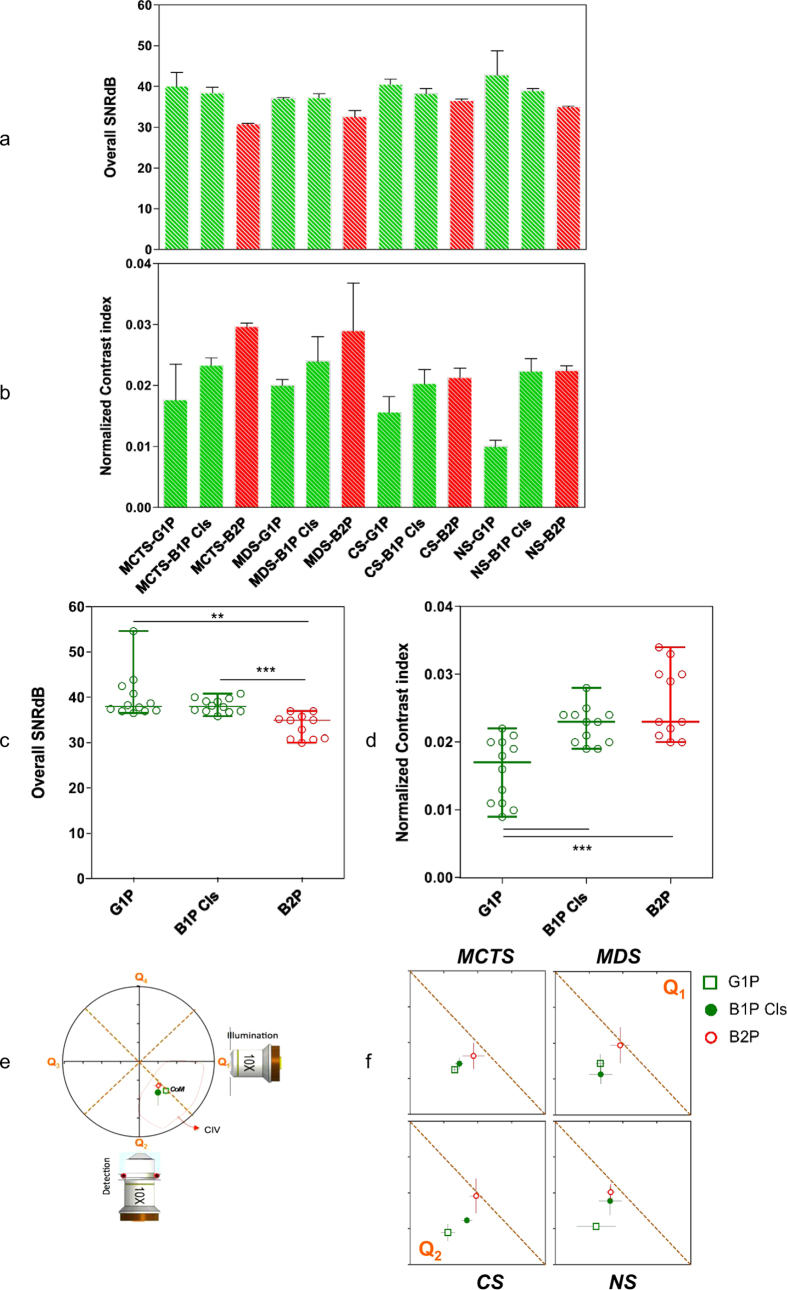
SNR, contrast and penetration depth comparison for various TMs imaged by different light sheet illumination modalities. SNR mean values. (**a**) for each TM and each modality in decibels (dB). NCI mean values (**b**) for each TM and each modality. (**c**) SNR mean values for each modality all TMs taken together and (**d**) NCI for each modality all TMs taken together **P < 0.01, ***P < 0.001 (t-paired test with a Welch correction and confidence interval of >95%). All error bars are the s. d. (**e**) Description of the graphical representation of the quadrant distribution and the CoM of the Contrasted Imaging Volume (CIV). The position of the CoM is referenced to the center of zenithal projection of the equatorial slice of the imaged TMs. The placement of the excitation and emission objectives shows the orientation of the images in relation to optical paths. (**f**) Graphical representation of CoM in the TMs for each modality. CoM is plotted in function of optical path axes (detection path vertical axis and illumination path horizontal axis). The dashed line represents the boundary between quadrant Q1 and Q2.

Firstly, we observed that TMs selected for this study, indeed, differed in term of nuclear size, cell density and level of heterogeneity (Fig. 3). In addition, we saw that they behave differently with respect to the different light sheet illumination modalities. As shown in Fig. 4a,b the SNR was relatively stable across TMs and light sheet illumination modalities, being in all cases, larger than 30 dB. Nonetheless, B2P generally displayed lower SNR than linear modalities (Fig. 4c). To achieve such SNR levels over the chosen FOV, a high-power laser was needed, typically 500 mW (measured behind the illumination objective), corresponding to the maximum power of the available laser source. The latter was thus the limiting factor in obtaining an SNR identical to that of linear modalities (Fig. 4c).

In terms of the NCI, variability in behavior was notably greater across TMs, resulting in a 73% difference in NCI values at the extremes (Fig. 4d). The graphs show maximum NCI for B2P regardless of the TM imaged. In the case of CS and NS, B1P Cls behave like B2P, providing a similar level of NCI. Furthermore, depending on the illumination modalities, similar behavior was found on the one hand for MCTS/MDS, and on the other hand for CS/NS (Fig. 4b). Results showed that NCI for CS/NS was typically lower than for MCTS/MDS.

In the 3D PSF measurements, results obtained were similar compared to those in the first stage of experiments. No significant differences in behavior appeared among the TM investigated herein. (Supplementary Fig. 6).

In light sheet microscopy, two types of penetration depths are generally distinguished: one along the illumination direction (lateral) and the other along the detection direction (axial) (Fig. 4e,f). To determine the penetration depths of these two directions we look at a *z-x* section of the TMs, taken at the equator of the TM. This *z-x* section, is then divided in four quadrants. Quadrants Q1 and Q4 reflected the penetration depth of the detection path, while quadrants Q2 and Q3 reflected that of the illuminating beam (Fig. 4e). To compare the penetration depth achieved by the different illumination modalities, we then defined the CoM of the contrasted-imaged volume (CIV; Supplementary Note 3) and plotted it on a chart corresponding to the intersection between Q1 and Q2. This representation enabled us to clearly visualize and compare the CoM as well as the penetration depth efficiency along both optical paths (illumination and detection). As expected, the CIV covered approximately half (30 to 60%) of quadrants Q1 and Q2, corresponding to the intersection between the well-illuminated and well-detected regions. Regardless of the TM imaged, we observed that among the illumination modalities, B2P displayed the most symmetric profile with respect to its CoM and offered better overall penetration depth (Fig. 4f). B1P Cls achieved better penetration depth for CS/NS than for MCTS/MDS, whereas G1P performed more poorly for CS/NS than MCTS/MDS. Interestingly, B1 Cls behaved like B2P in the case of NS (Fig. 4f). Strikingly, the ability of the B1P Cls illumination modality to image in-depth within TMs seemed to correlate directly with the size of cell nuclei.

**Figure 5:**
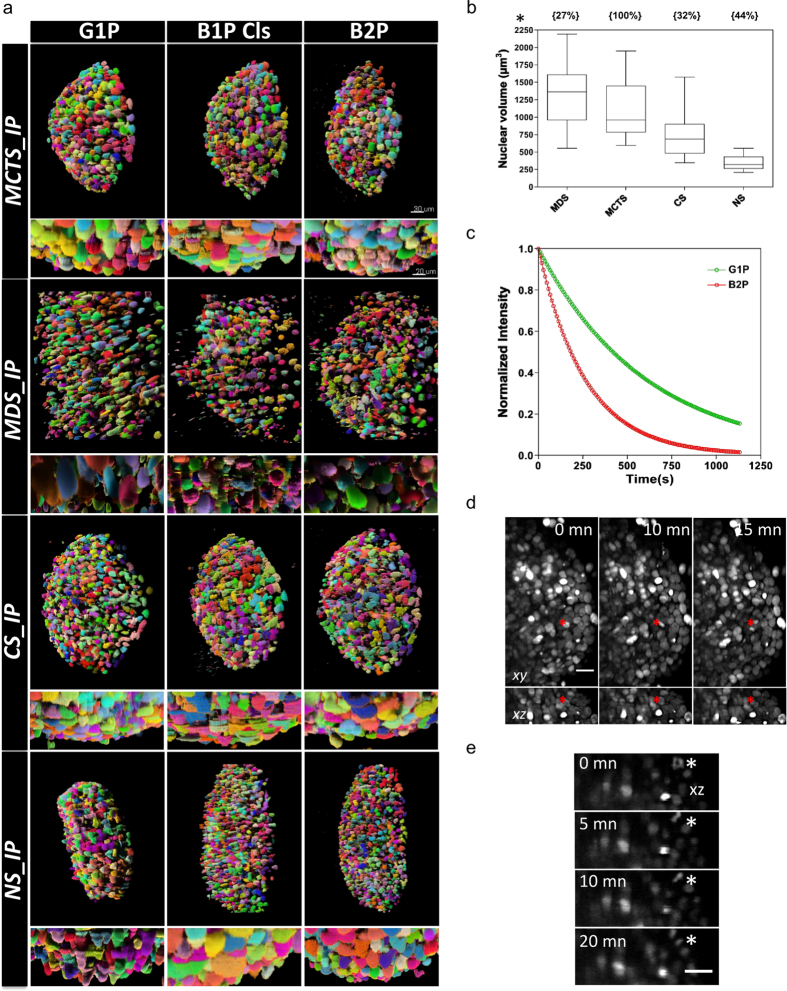
(**a**) Surface rendering of 3D stack segmentation masks in which each color indicates a segmented object, obtained for different TMs imaged by different light sheet illumination modalities (scale bar 30 μm). Zooming axial views (scale bar 20 μm). (**b**) Quantitative analysis of nuclear volume for different TMs. Box plots of nuclear volume indicating the lowest quartile, the median value and the top quartile. The density for each TM is indicated in brackets above the box plots. All error bars are the s. d. (**c**) Normalized photobleaching curves for MCTS stably expressing H2B-mCherry with continuous excitation with G1P or B2P. (**d**) Maximum projection along (xy) and (xz) of a MCTS stably expressing H2B-mCherry acquired with B2P at the indicated times. Stacks of 100 slices were recorded every 5 minutes with a slice spacing of 1 μm. The red asterisks show a dividing cell. Scale bar, 20 μm. (**e**) xz enlarged region of the MCTS at the indicated times. The progress of mitosis can be seen in one cell (asterisk).

To verify this hypothesis, we measured the nuclear volume and the density of cells for each TM imaged in this study. As shown in Fig. 5b, TMs showed great differences according to both parameters. For example, NS contained a high density of cells with a relatively homogeneous distribution of small, relatively homogeneously sized nuclei, while MDS rather displayed a low density of cells having very heterogeneously sized nuclei, three times bigger on average. This analysis supported the existence of a correlation between the depth penetration performance of B1P Cls and the size of TM nuclei, but apparently not between depth penetration performance and cell density.

### Applicability

Even though our analysis provides meaningful information about the performance of different LSFM implementations for 3D TM imaging, it does not however, predict their concrete applicability, notably in terms of suitability for image processing and analysis. Indeed, while TMs offer an appealing opportunity to study a wealth of biological questions at the tissue level, most of the available quantitative parameters require that all structures of interest be properly segmented. This is a task that can no longer be tackled manually given the sheer amount of data routinely produced by LSFM systems, and now requires instead efficient computerized image processing algorithms.

Consequently, a comprehensive comparison of LSFM systems should therefore also question whether imaging performance effectively correlates with the performance of computerized analysis methods. We illustrate this issue, specifically, with segmentation of cell nuclei in 3D images of TMs, particularly challenging due to high cell density and variability in nuclear size and shape. Segmentation is currently a very active field of algorithmic research in the bioimage informatics community. We selected one such open-source software, namely TGMM^40^,^41^ designed to track nuclear-labeled cells in 3D images, and demonstrating good performance on datasets from different developing embryos studied with LSFM. Figure 5a illustrates the usability of the TGMM software on 3D TMs imaged with the different illumination modalities. Visual inspection by a human expert of the segmented nuclear shape showed that TGMM fails to correctly segment densely packed cell nuclei in TMs imaged with G1P, due to the unusually elongated shapes produced by the algorithm. In contrast, TGMM performed much better for B2P, producing well-rounded and separated cell nuclei regardless of the TM imaged, while results obtained via B1P Cls lay somewhere in between the two. These findings suggest that B2P presents the greatest applicability in TM imaging from an image analysis perspective, although this advantage is won at the cost of the high laser power required for TM imaging.

The versatility of TMs for cell biology studies naturally extends to live imaging studies at the single cell level. While all LSFM support time-lapse imaging, they vary greatly in terms of photobleaching and photodamage (PB). Typically, B2P requires a higher laser power than its counterparts, thus potentially limiting its applicability to fixed (or short-term) imaging of TMs. It is indeed well known that mammalian cells are significantly less compliant to high- intensity illumination than organism models such as *Drosophila melanogaster or Caenorhabditis elegans* imaged under a B2P regime^26^,^42^. We measured this effect by examining, on the one hand, the photobleaching rate (Fig. 5c) and on the other, the overall health of cells by monitoring cell division events (Fig. 5d, e) within MCTS stably expressing mCherry-H2B. We found a more pronounced and twice faster photobleaching of mCherry-H2B with the B2P modality (decay-time 27.6 s) than with the G1P modality (decay-time 63.6 s; Fig 5c). Meanwhile, we were only able to image a few cell division events shortly after placing the MCTS under the microscope, and could not find any dividing cells after about one hour of imaging, indicating a cell cycle arrest due to photodamage. Additionally, we noticed several cases of structural breakdown in the TM during B2P imaging of MCTS, not unlike that typically achieved by laser ablation techniques. Taken together, these results confirm that the use of B2P for 3D TM live imaging in these experimental conditions was limited by photobleaching and photodamage, and that one-photon excitation (perhaps aided by confocal line detection, i.e. B1P Cls) should be preferred in such contexts.

## Discussion

Comparison analyses are fundamental tools that sharpen our knowledge and play key roles in establishing methodological guidelines and in driving continuous improvements. However, performing these analyses in a way that is unbiased, fair, transparent, practical and acceptable by the scientific community remains a major challenge. Here, we have developed the first systematic, quantified method to assess performance of different LSFM implementations for 3D TM imaging. As criteria for comparison, we took into account four typical measures related to image quality and specifically designed for LSFM and 3D TM imaging: signal-to-noise ratio, image contrast, spatial resolution and penetration depth. Our analysis has enabled us to clearly point out the strengths and weaknesses of each technique. For example, we found that the illumination modalities G1P as well as B1P Cls, give fairly good results regardless of the TMs studied, although they display a large variation of the axial PSF (Supplementary Fig. 6) impacting on its resolution. The gain in contrast offered by the B1P Cls modality is significant for TMs displaying small nuclei (CS and NS), while it is less evident for TMs with large nuclei (such as MCTS/MDS). Contrast, resolution and penetration depth emerged as the main strengths of B2P outperforming other illumination modalities with no significant differences across TMs studied herein.

Our experiments show that the proposed metrics are concise, interpretable, and reliable enough to enable a fair and unbiased comparison. Table 2 summarizes the observations from our study. As expected, there is no clear winner among the various techniques tested, and the choice of modality typically depends on the application at hand. G1P gives the best SNR but lacks on contrast and sectioning capabilities. In comparison, B1P Cls provides fair SNR but a larger FOV and better sectioning capabilities. Finally, B2P provides the largest FOV and a highly homogeneous *x,y,z* resolution, but it does not performs as well in terms of SNR. Overall B1P Cls seems to provide the best compromise among all measured criteria if the uniformity of the sectioning capabilities are not demanding for the measurements. In terms of PB for *in vivo*, long-term imaging, G1P and B1P Cls provides similar results while in B2P presents a major issue and further technical efforts are necessary to take this technique into living imaging.

A possible route to make B2P compatible with 3D TM long term imaging could be by using or developing specific new lasers completely adapted and optimized for this illumination modality. For efficient interaction, the laser wavelength must be carefully chosen to match the peak of the two-photon absorption cross section of the used label. In addition, based on calculating the efficiency of the nonlinear generated signal, it is possible to optimize the laser parameters for multiphoton imaging, following a figure of merit (FOM) based on average power *P*_*average*_, pulse duration *τ*, pulse repetition rate *R*^43^:


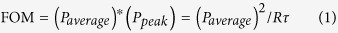


Thus, one can increase the two-photon excitation fluorescence signal by increasing either the peak or the average power of the excitation beam. This is the same as saying that, for a given average power and pulse duration, two-photon excitation fluorescence efficiency can be increased by reducing the repetition rate of the laser^44^. Indeed, the FCPA Jewel D1000 used in ref. 44 in comparison with the Ytterbium laser used in this work, should produce about 1000 times more two-photon emission for same average power.

Along the same lines, further improvements in the Bessel Cls modality can be implemented, as, for example, the sectioned Bessel beams already proposed^45^ that would, in principle, improve uniformity on the axial PSF in all of the TMs and thus overall image quality of the TMs.

Overall, it seems clear that current LSFM implementations still pose significant challenges in terms of computerized data analysis, leaving room for further improvement both in imaging and analysis. Finally, it is worthwhile to emphasize that 3D TM imaging is an emergent and powerful field attracting considerable attention to its potential to deliver higher quality information data. However, retrieving this high quality information currently means striving for continuous improvement of existing imaging tools, but also developing new, specific ones.

## Methods

### Cell lines

HCT116-H2B–mCherry cells were cultured in DMEM +GlutaMAX (Dulbecco’s Modified Eagle Medium; Gibco) supplemented with 10% fetal bovine serum and 1% penicillin–streptomycin (Pen Strep; Gibson), and maintained at 37 °C and 5% CO_2_ in an incubator. MCF10A (ATCC^®^ CRL-10317™) were cultivated in DMEM/F12 supplemented with 5% fetal bovine serum, 1 μm/ml insulin, 0.5 μg/ml hydrocortison, 20 ng/ml EGF and 100 ng/ml cholera toxin.

### Isolation and culture of neonatal cardiomyocytes

Neonatal CMs were isolated from 2-days-old Sprague-Dowley rats. Following heart digestion with 0.1% collagenase type II (Sigma), cells were resuspended in complete medium (Ham’s F-12 medium, 10% fetal calf serum, 10% horse serum and 1% penicillin-streptomycin) and then incubated for 2 h at 37 °C, allowing the selective attachment of non-myocytes. CM-enriched suspensions were then plated at a density of 50000 cells into 5 ml in complete medium.

### MCTS production

To produce the MCTS, we used the centrifugation method described in Lorenzo *et al*.^11^ thus obtained a single spherical MCTS with a readily reproducible size (coefficient of variation less than 10%). MCTS were prepared in 96-well plates that were coated with 20 mg/ml polyHEMA (Sigma). Cells were plated at a density of 600 cells/well in 100 μl cell culture medium then centrifuged to enable MCTS formation. When necessary, 0.5 μm fluorescent beads (Invitrogen Inspeck Green 505/515 100% relative intensity) were added to the cells before centrifugation. After 4–5 days growth, MCTS of 400 μm in diameter were collected, washed three times with PBS and then fixed with 10% neutral buffered formalin (Sigma–Aldrich) at room temperature for two hours.

### MDS and CS production

To produce the MDS and CS, we used agarose 3D Petri Dish^®^ made in 12-series micromolds (sigma-aldrich). The desired cells in suspension (1.5 × 10^6^ cells in 190 μl and 3.5 × 10^5^ cells in 75 μl of culture medium respectively for MDS and CS) were added drop-wise to the center of the 3D Petri Dish^®^. When necessary, 0.5 μm fluorescent beads (Invitrogen Inspeck Green 505/515 100% relative intensity) were added to the cells. After incubation at 37° in 5% CO_2_ atmosphere for indicated time, MDS and CS of 400–450 μm in diameter were collected by pipetting, washed three times with PBS and then fixed with 10% neutral buffered formalin (Sigma–Aldrich) at room temperature for two hours.

### NS production

Spinal progenitor stem cells were obtained from spinal cord of E12.5 mouse embryos. Briefly, spinal cords were dissociated in ice-cold HBSS solution and transferred to pre-warmed DMEM/F12. Single cell suspension was obtained by mechanical dissociation of pooled tissues. Cells were centrifuged at 1500 rpm and resuspended in pre-warmed cell culture medium freshly prepared (DMEM/F12 supplemented with 1.5 mM Putrescine, 5 mM Hepes, 3 mM NaHcO3, penicillin-streptomicin, 1x B27 supplement, 1x N2 supplement, 1x ITSS, 5 ng/ml FGF, 20 ng/ml EGF, 30% glucose). Cell culture medium was refreshed every 2–3 days by adding 10% fresh medium. Spinal progenitor stem cells grown in suspension form “neurospheres” which were dissociated every 2 weeks using 0.25% Trypsin solution, diluted 1/3 and transferred to fresh cell culture medium.

### Animal experimentation statement

All animal experimentation was conducted in accordance with the European and the French regulation procedures and was approved by the local animal ethic committee (CEEA 122) and the French Ministry as required. Animals (mice, rats) was obtained from labelled furnishers and housed at the UMS006-CREFRE zootechny facilities. This work doesn’t involve living animal experimentation. After sacrifice using validated methods, the tissue samples was removed and collected.

### Propidium iodide (PI) staining

After formalin fixation, TMs were incubated at room temperature for 30 min with a solution of 0.5 ml PBS containing 10 μg/ml *RNase* A and 20 μg/ml PI and then washed three times with PBS. For imaging fixed TMs were embedded in 1% agarose (Euromedex Low Melting Point) in 50 μL capillary pipettes (Hirschmann Laborgeräte Ringcaps).

### Nuclear volume measurements

To determine the nuclear volume of cells, 30 isolated nuclei were cropped from Z stacks acquired with the B2P modality for each of imaged TMs. 3D watershed segmentation plugin available from FIJI was then applied using automatic seed detection. The parameter “Radius for automatic seeds” was set according to the respective TMs. Finally the volume of each segmented nucleus was obtained using the 3D ROI manager plugin available from FIJI.

### Density cell determination

Nuclei number (Nnuc) present in Regions Of Interest of 80*80 μm (ROI_Surface_) was determined by manually counting. The 2D surface of nuclei (2Dnuc) was then determined from nuclear volume measurement (see above). Finally, the density cell was calculated as following^46^: (*100)/ROI_surface_.

### Cell nuclei segmentation and 3D visualization

To perform the cell nuclei segmentation of TM images, we used the TGMM software as described in Amat *et al*.^40^. The “*persistenceSegmentationTau*” was fixed at 20 while the “*backgroundThreshold*” parameter was set according to the respective TM images. No further adjustments were made. Using the provided “*ExtractSegmentationMatrix*” matlab code, we generated a 3D segmentation mask. tiff file from the output. svb. The surface rendering of 3D segmentation result was then obtained using Imaris^®^.

### Photobleaching measurement

MCTS images were taken every 10 s using a 500 ms exposure with continuous illumination for z tps. Calculated normalized photobleaching kinetics PB were obtained by dividing the mean- corrected fluorescence intensity I in each frame by the first frame: PB = (I_(n)_ − I_BKG_)/(I_(1)_ − I_BKG_). In order to analyze the bleach rate of image sequences, we used the PixBleach pluging^47^ available from FIJI.

### Timelapse imaging

MCTS live-imaging experiments were performed with HCT116 cell line stably expressing the histone H2B fused to the mCherry fluorescent protein; MCTS were put into a culture medium-filled physiological chamber inside a phytagel container as described in Desmaison *et al*., 2012. An incubator was fitted above the LSFM to control the temperature and CO2 concentration around the sample (PECON controllers). mCherry was excited with the B2P light sheet using a 1040 nm laser (Mikan Amplitude Systemes). Leica 10x/0.3 numerical aperture (NA) 20x/0.5 water immersion objectives were used respectively for excitation and emission. Emitted light was collected through a 594-nm long-pass detection filter (Semrock) and a Hamamatsu Orca Flash 4.0 sCMOS camera (binning 2, lateral pixel size in the acquired images, 0.65 μm). Image stacks of 200 planes encompassing a half of an MCTS with an axial step size of 1 μm were acquired at 5 min intervals for several hours.

## Additional Information

**How to cite this article:** Andilla, J. *et al*. Imaging tissue-mimic with light sheet microscopy: A comparative guideline. *Sci. Rep.* **7**, 44939; doi: 10.1038/srep44939 (2017).

**Publisher's note:** Springer Nature remains neutral with regard to jurisdictional claims in published maps and institutional affiliations.

## Supplementary Material

Supplementary Information

Supplementary Movie S1

## Figures and Tables

**Table 1:** 3D TM imaging with LSFM, a comparison.

Modality	Tissue Mimic	Fluorescent label	Exc. regime/λ	Objective_ill/NA	Objective_det/NA	FOV	LS thickness	Exposure time	laser power	reference
Conventional LSFM										
SPIM	Fixed BxPC3 MCTS(⦸ 140 μm)	Draq5	1p@633 nm	10X/0.3	40X/0.8	N/D	N/D	N/D	N/D	Verveer P.J.^8^
	Live HCT116 MCTS (⦸ 400 μm)	H2B-HcRed	1p@595 nm	10X/0.15	10X/0.3	N/D	3 μm	500 ms	N/D	Lorenzo C., 2011^11^
	Live MDCK cell aggregates (⦸ 50 μm)	GFP-Actin, H2B-YFP, Syto61	1p@488 nm/543 nm/633 nm	5X/0.16	40X/0.8	N/D	2–4 μm	N/D	N/D	Pampaloni F, 2014^48^
Inverted microscope-SPIM	Live CHO MCS(⦸ 100 μm)	pAcGFP1-Men or acridine orange	1p@488 nm/470 nm	N/D/0.08	10X/0.3 or 20X/0.5	400 μm	10 μm	100 ms or 1 s	52 mWcm-2	Bruns T, 2012^49^
	Live Coculture HepG2/Huvec MCS(⦸ 250 μm)	Cell tracker green, DsRed	1p@470 nm/545 nm	N/D	20X/0.5 or 40X/0.6	300 μm	9–10 μm	N/D	N/D	Patra B, 2014^50^
DSLM	Fixed BxPC3 MCTS (⦸ 100 μm)	Draq5	1p@633 nm	N/D	40X/0.8	N/D	3.5 μm	100 ms	30 nWμm-2	Swoger J, 2013
	Live or fixed neural aggregates (⦸ 100–300 μm)	GFP, TH, bIITubulin aN/D TO-PRO-3 NucView & MitoView	1p@488 nm/568 nm/647 nm	4X/0.13	16X/0.8	819*819 μm	N/D	N/D	N/D	Gualda E, 2014^12^
	Fixed MCF7 MCTS in coculture with fibroblast	RFP, anti-vimentin antibody	1p@473 nm/561 nm/642 nm	4X/0.13	10X/0.3	N/D	N/D	N/D	N/D	Gualda E, 2015, 2016^51^,^52^
Advanced LSFM										
SPIM										
IML-SPIM	MCF10A MCS (⦸ 150 μm)	H2B or connexin 43 -PAmCherry	1p@405 nm/561 nm	N/D	40X/0.8 or 100X/1.1	80*80 μm	4 or 1.8 μm	2–3 min	0.06 kW cm-2/5–12 kWcm-2	Zanacchi F, 2011^53^
2PE-SPIM	Fixed MCF10A Acini	Hoechst 33342	2p/750 nm	10X/0.3	20X/0.5	292.81 μm	N/D	N/D	54.94 kWcm-2	Lavagnino Z, 2013^54^,^55^
DSLM										
sectioned Bessel beams	Fixed CT26 MCS (⦸ 250 μm)	Phalloidin-Alexa488	1p@488 nm	N/D/0.4	N/D/0.8	100 μm	N/D	N/D	N/D	Farbach F., 2013^17^
2p Bessel	Fixed CT26 MCS (⦸ 250 μm)	Phalloidin-Alexa488	2p@920 nm	N/D/0.4	40X/0.8	562 μm	N/D	N/D	N/D	Farbach F., 2013^54^
Airy beam	Fixed ACHN MCS	Alexa488-WGA	1p@488 nm/532 nm	20X/0.42	20X/0.4	346 μm	0.865 μm	10–50 ms	30–300 μW BFP; 70 Wcm-2	Vettenburg T, 2014^20^
Lattice (Single plane)	*Live Mouse ESC MCS (⦸ 35 μm)	Halo-Tag-TMR-Sox2	1p@560 nm	Special optics/0.55	25X/1.1	100 μm	N/D	10 ms	900 μW	Chen BC, 2014^28^
WAOSPIM	Fixed HCT116 MCTS (⦸ 400 μm)	HcRed-H2B/mCherryH2B	1 P@595 nm	10X/0.15	20X/0.5	N/D	3 μm	100–500 ms	N/D	Jorand R, 2012^21^Masson A, 2015^55^

⦸ diameter N/D Not Defined * < 100 μm.

**Table 2:** Summary of the metrics used to evaluate the different modalities and TMs acquisitions.

		MCTS	MDS	CS	NS
*G1P*	SNR	++	+	++	++
	CI	.	.	−	−−
	FOV	.	.	−	−
	PB	+	+	+	+
	PSFz	.	.	.	.
*B1P cls*	SNR	++	+	+	+
	CI	+	+	.	.
	FOV	.	.	.	+
	PB	+	+	+	+
	PSFz	. (*)	. (*)	. (*)	. (*)
*B2P*	SNR	−−	−−	−	−
	CI	++	++	.	.
	FOV	.	+	+	+
	PB	−	−	−	−
	PSFz	+	+	+	+

++Very good, +Good, Fair, − Poor, −− Very poor. (*) The behaviour of the digital filtering is not uniform in the whole volume introducing a big variability in the expected value.
