# Supplementary material for: Imaging tissue-mimic with light sheet microscopy: A comparative guideline
Source: Sci Rep. 2017 Mar 21;7:44939. doi: 10.1038/srep44939 (PMC5381005; doi:10.1038/srep44939)
Supplement: Supplementary Information [file srep44939-s1.pdf]

Imaging tissue-mimic with light sheet microscopy: A comparative guideline

Jordi ANDILLA, Raphael JORAND, Omar E. OLARTE, Alexandre C. DUFOUR, Martine CAZALES, Yoann LE MONTAGNER, Romain CEALATO, Nicolas RIVIERE, Jean- Christophe OLIVO-MARIN, Pablo LOZA-ALVAREZ and Corinne LORENZO

**Supplementary Figure 1**	Schema of the Melopee instrument adapted for TM measurements
**Supplementary Figure 2**	Single Multimodal-LSFM setup
**Supplementary Figure 3**	Beam profiles
**Supplementary Figure 4**	MCTS imaged with the six illumination modalities
**Supplementary Figure 5**	Image analysis framework
**Supplementary Figure 6**	TM 3D PSF
**Supplementary Table 1**	Simplified specifications of the Melopee instrument
**Supplementary Table 2**	Main components of Multimodal LSFM setup
**Supplementary Table 3**	Experimental imaging parameters for the first stage of experiments
**Supplementary Table 4**	Experimental imaging parameters for the second stage of experiments
**Supplementary Note 1**	MFP measurements
**Supplementary Note 2**	Single Multimodal LSFM setup
**Supplementary Note 3**	Image analysis framework
**Supplementary references**	
**Supplementary Software Icy**	
**Supplementary movie**	Tutorials of the protocols

Supplementary Note 1: MFP measurements

We combine spectroscopy, polarimetry and scatterometry within a single instrument to study the propagation of an electromagnetic wave within the TMs. Due to the huge amount of data involved in hyperspectral measurements (more than 5,000 wavelengths), tensorial quantities were used to store the scattering data in a hypercube. The angular and spectral dependences were used to obtain the Bidirectional Reflectance Distribution Function (BRDF) and the Bidirectional Transmittance Distribution Function (BTDF)[1]. By doing so, the polarimetric bidirectional reflectance and transmittance tensors are the tensorial representations of the spectral polarimetric BRDF and BTDF. They are defined as the ratio of the polarized reflected or transmitted radiance tensor to the polarized incident irradiance tensor with discretized angles and wavelengths. Multiple scattering in the sample tends to modify the light polarization by randomizing the polarization state of the scattered light. However, the incident polarization may be maintained over distances much larger than the photon Mean Free Path (MFP). Thus, measurements of the depolarization provide relevant information about how light propagates within MCTS. One can derive and directly compute the Degree of Linear Polarization tensor from the polarimetric bidirectional reflectance and transmittance tensors. For an incident light with a linear polarization state, these tensors give useful information about the complex scattering processes. Based on supercontinuum sources, we developed a new, fast, non-contact, label-free and highly spectral and angular resolved technique to measure the hyperspectral (from visible to near infrared), polarimetric and angular scattered-light intensities of MCTS. This technique provides a specific fingerprint resulting from complex interactions between white-light and MCTS. Our method exploits an extension of the instrument displayed in Supplementary Figure S1. The instrument has been fully detailed in Ceolato et al.2 and only simplified specifications of the instrument are summed up in Supplementary Table S1.

Supplementary Note 2: Single Multimodal SPIM

- Optical setup

The optical setup we have implemented is shown in Supplementary Figure 2. The xy plane is defined as the image plane of the collection lens, and the z-axis as the direction orthogonal to that plane. The excitation beam propagates along the *x*-axis, and the light sheet is generated by the excitation objective (EO) in the *xy* sample plane. For DSLM modalities, the light sheet is generated by scanning the beam along the *y* direction using one galvanometric mirror (*y* GM). Another galvanometric mirror (*z* GM) is employed to move the light sheet in the z direction for focusing purposes. A telescopic system composed of a scanning lens (SL) and a tube lens (TLE) is placed behind the GM and is such that the EO back focal plane and the GM planes are conjugated. The fluorescence generated is collected with a water-dipping infinity-corrected objective (CO) that is set at an optimum imaging distance from the light sheet (equal to the working distance of the objective). A regular tube lens (TLC), providing a 25· magnification, is used to form an image of the fluorescent structures on the sCMOS sensor.

Two lasers provide excitation light for the different modalities: a Yb-based femtosecond laser emitting at 1040nm (fs-NIR) for nonlinear, and a continuous wave Diode-Pumped Solid-State laser emitting at 532nm (CW-Visible) for linear excitation.

The system can be configured to work in one of the following six modalities: i) linear SPIM (G1P CL), DSLM with ii) Linear Gaussian beams (G1P), iii) nonlinear Gaussian beams (G2P), iv) linear Bessel beams (B1P), v) linear Bessel beams and confocal line scan (B1P Cls), and, vi) nonlinear DLSM with Bessel beams (B2P).

To switch between the linear and nonlinear modalities, a mirror positioned on a flipping mount (FM) is employed to direct either the CW-Visible or the fs-NIR to the system. To select among the modalities corresponding to the beam shapes, a translational stage (TS) can be set to three different positions: 1) G1PCL, where a cylindrical lens (CL) is

inserted, 2) Gaussian modes, where the beam path is unaffected, and 3) Bessel modes, where an Axicon (Ax) and a lens (AxL) are inserted.

In the first set of experiments, the magnification of the telescopic system formed by SL and TLE and the laser beam waist were set to adjust the length of the Bessel modes to 480 μm of field-of-view (FOV). In these conditions, the Gaussian modes covered a FOV of approximately 250μm. In the second set of experiments the optical system was set to obtain a global FOV of bout 200 μm in all modalities as described in supplementary table 4.

The B1P Cls modality requires setting the rolling shutter of the sCMOS camera to synchronize with the position of the beam when using the linear Bessel beam. A given line of the pixels is activated during the passage of the central lobe of the Bessel beam through its active area. This means that the activation rate of each line corresponds to the scanning rate of the GM and that the opening time is defined by the size of the central lobe of the beam. Finally, maximizing the acquired signal sets the scanning amplitude of the GM as well as the phase between the GM and the rolling shutter.

The samples are held by a custom-made capillary sample holder (SH) that is attached to a three-axis motorized stage (*x, y* and *z*) and a rotation stage that turns the sample around the *y* axis (angle ø). The SH is located inside a custom-made immersion chamber filled with a physiological fluid (PBS). The chamber was made by machining a solid cube of Teflon™ and is optically accessible from three faces through glass windows made with coverslips (N= 1.5, 170 μm thickness). The CO enters the chamber from a hole machined on the remaining face of the cube. The metallic body of the objective is held in the chamber using an O-ring that seals the chamber preventing PBS leakage. A list of the main components of the system can be found in Supplementary Table 2.

- Fitting the beam profiles

To obtain a quantitative comparison of the different modalities we have used an approach consisting of analyzing the images of each beam intensity distribution (Figure 2) and measuring the changes in both intensity and width of the beam. To do so, the images were sliced in vertical lines (y direction) and the resulting accompanying intensity profiles were individually analyzed. In order for the intensity of different beams to evolve with propagation, the maximum value of each profile was extracted and represented as a function of the position along x. Moreover, to capture the evolution of the width of the beam as it propagates, a fit of each vertical line was performed according to the following functions:

$$f(y) = A + Be^{-\frac{(y-C)^2}{2D^2}}, \qquad 1$$

for both linear and nonlinear Gaussian modalities,

$$f(y) = A + Be^{-\frac{(y-C)^2}{2D^2}} + E \cdot sinc\big(F \cdot (y-C)\big), \qquad 2$$

for B1P, and,

$$f(y) = A + Be^{-\frac{(y-C)^2}{2D^2}} + E \cdot sinc^2\big(F \cdot (y-C)\big), \qquad 3$$

for B2P.

In both Gaussian modalities the D coefficient in eq.(1) accounts for any change in the width during the propagation of such beams. On the other hand, in the Bessel modalities, the Gaussian term accounts for the average broadening of the beams, while the sinc/sinc^2 term accounts for the non-diffracting component of the beams during

propagation, see eqs.(2-3). Coefficients B and E of eqs. (2-3) represent the strength of each of these components, whereas D and F give information about their width. For the linear DSLM-Bessel modality, profiles were fitted to a sinc instead of a $sinc^2$ function, as we thus found better and more reliable results. This function may not have physical significance, as it allows negative values, but, normally, the Gaussian component helps to compensate for and alleviate this condition. For the Gaussian modalities, widths are reported as the full-width-at-half-maximum (FWHM) calculated from the D coefficient in eq.(1) as $FWHM_G$=2.35D, whereas for Bessel modalities, $FWHM_B$ was measured numerically from the fitted functions.

Supplementary Note 3: Image analysis framework

Supplementary Figure 5 summarizes the workflow. Both TM models are first imaged in 3D with an LSFM modality (Supplementary Fig. 5a, b). With TM labeled nuclei dataset, protocol P1 is used to automatically detect the TM and crops a 3D sub-volume centered on it (Supplementary Fig. 5c). Protocol P2 is then used to measure the SNR, Contrast Index (CI), Normalized Contrast Index (NCI) and Contrasted Imaging Volume (CIV) on the extracted sub-volume (Supplementary Fig. 5c). The SNR is obtained as a ratio between the dynamic range of sequence, which measures the gap between the brightest objects and the darkest areas in a 3D image in terms of intensity, and the standard deviation of the noise present in the 3D image. These two quantities are evaluated as follows:

- The dynamic range is obtained as the difference between the 99.5% and the 0.1% quantiles of the voxel intensities. Compared to the approach that defines the dynamic range as the difference between the maximum and the minimum of the

voxel intensities, our quantile-based method is more robust with respect to the presence of erroneous voxel intensity measurements.

- The method proposed to evaluate the standard deviation of the noise follows an approach proposed by Starck & Bijaoui[6], which consists of (i) computing a low-pass filtered version of the 3D image by applying an isotropic Gaussian kernel with a standard deviation parameter set at 1.5 pixels, (ii) subtracting this low-pass filtered 3D image from the original one to extract its high-frequency content, which corresponds mostly to noisy data. The standard deviation of this residual is then evaluated through a rank estimator. It is important to note that the low-pass filtered version of the 3D image involved in this computation should not be understood as a denoised version of the input: in particular, the Gaussian kernel may introduce significant artifacts close to the object edges in the image. However, in homogeneous areas, the Gaussian kernel is efficient in separating the noise from the signal of interest.

With this definition, the SNR measure is invariant with respect to linear contrast transformations.

The CI is measured using a 3D contrast map representing the local intensity variance computed for each voxel (Supplementary Fig. 5d). To define the contrast index, we first built a contrast index map, which evaluates the local contrast in the vicinity of each voxel of the 3D image. More precisely, the value of the contrast index map at a given voxel X is defined as the standard deviation of the voxel intensities in a Gaussian window shaped with a standard deviation parameter set at 1.5 pixels. All the local contrast measures are then aggregated into a global index, by taking the square root of the mean of the square of the contrast index map. This approach follows the one proposed in Wang & Mean[7] to characterize image contrast information. With this

definition, the contrast index is not invariant with respect to linear contrast transformations. We therefore define a normalized version of the contrast index NCI, that is: the normalized contrast index is obtained as the ratio between the contrast index and the dynamic range measure introduced in relation to SNR evaluation.

To determine CIV, *xz* cross-sectional images are used as an indication of the apparent penetration depth of the imaging systems. The CIV is then estimated in 3 steps: (i) the equator volume of the TM is extracted from the 3D contrast map (Supplementary Fig. 5d) coplanar with both illumination and imaging axes, with a thickness equaling 20% of the TM; (ii) The extracted volume (Supplementary Fig. 5e) is projected into a 2D image using Maximum Intensity Projection (MIP); (iii) Finally, quadrants are delineated on the MIP, and the percentage of area displaying more than 5-20% of local contrast in each quadrant is measured (Supplementary Fig. 5f).

With the TM_Beads dataset, protocol P3 is used to characterize the PSF in 3D in the entire imaged volume. The protocol starts with a wavelet-based spot detection used to detect the positions of beads within the 3D volume and then extracts a sub-volume of six times the size of the expected 3D PSF. 2D Gaussian function is then fitted to the central profile of each sub-volume along the three axes (*x, y, z*) and the FWHM is then computed from the standard deviation of the Gaussian fit providing the actual size of the 3D PSF. The values of FWHM of the PSF in the entire volume in 3D are then listed in a file.

The analysis of the results obtained from the P3 protocol provides a pair of values describing the typical resolution (mode) and the dispersion of the values (standard deviation) of FWHM for each modality studied and axis analyzed. The shape of the distribution is obtained by computing the histogram of the list of the FWHM values. The

step size of the histogram was 0.3 μm and the outliers (values greater than 30 μm) were filtered to give a histogram with 100 bins.

The distribution was then fitted by a lognormal distribution which has a probability density function defined by:

$$f_X(x;\mu,\sigma) = \frac{1}{x\sigma\sqrt{2\pi}} e^{-\frac{(\ln x-\mu)^2}{2\sigma^2}}, x > 0 \qquad 4$$

where x is the independent variable (FWHM) and (μ, σ) are the fitting parameters. The Matlab™ curve-fitting tool (cftool) was used to determine the two fitting parameters. However, the distributions had an offset value on the independent variable due to the actual diffraction limit of the system. We therefore performed a gradient ascent maximization algorithm on the results obtained by the cftool taking the *x* offset as parameter ($x \rightarrow x - x_{offset}$) and the correlation coefficient *r* as the maximization merit function.

Supplementary References

1. Nicodemus, F. Directional reflectance and emissivity of an opaque surface. *Applied Optics* **4**, 767-773 (1965).
2. R. R. Alfano, S.L.S. Emission in the region 4000 to 7000 A via four-photon coupling in glass. *Phys. Rev. Lett* **24**, 584-587 (1970).
3. Champert, P.A. et al. White-light supercontinuum generation in normally dispersive optical fiber using original multi-wavelength pumping system. *Optics express* **12**, 4366-4371 (2004).
4. J. Dudley, G.G., S. Coen Supercontinuum generation in photonic crystal fiber. *Rev. Mod. Phys* **78**, 1135-1184 (2006).
5. Ceolato, R., Riviere, N. & Hespel, L. Reflectances from a supercontinuum laser-based instrument: hyperspectral, polarimetric and angular measurements. *Optics express* **20**, 29413-29425 (2012).
6. Starck J. L., M.F., Bijaoui A. Image processing and data analysis. *Cambridge University Press* (1998).
7. Wang, Z.a.B., A. Mean squared error: love it or leave it? A new look at signal fidelity measures. *Signal Processing Magazine* **29**, 98-117 (2009).

Supplementary Table 1: Simplified specifications of the Melopee instrument.

**Light-source**	Supercontinuum laser
**Wavelengths**	From 530 nm to 1070 nm
**Beam divergence**	< 5 mrad
**Spectral output power**	0.8 – 50 mW/nm
**Sensors**	CCD-based grating spectrophotometer
**Spectral resolution**	< 1 nm
**Dynamic range**	7

Supplementary Table 2: Main components of Multimodal LSFM setup

Item	Manufacturer/model	Notes
Fs-NIR Laser	Time Bandwidth/GLX-Yb	1040nm, 250 fs pulse-width
CW-Visible Laser	Coherent Inc. / Verdi V10	532nm
Galvo Mirrosr/y,z GM	Thorlabs/GVS002	Dual axis
Excitation objective/EO	Nikon/Plan Fluor 10x	NA=0.3, WD=16mm
Collection objective/ CO	Leica/HCX apo L20X	NA=0.5, WD=4mm
Axicon/Ax	Altechna/1-APC-1-G254	176 deg. apex angle
CMOS Detector	Hamamatsu/ ORCA-Flash 4.0 C11440-22C	
Sample holder/SH	Thorlabs/3-axis NanoMax, Physik instrumenteI M-116.2SH	Stepper motors for xyz translation and ϕ rotation.

Supplementary Table 3 : Experimental imaging parameters for the first stage of experiments

Modality	**G1P CL**	**G1P**	**G2P**	**B1P**	**B1PCls**	**B2P**
Objective Magnification	10x	10x	10x	10x	10x	10x
NA Illumination	0.0675	0.0675	0.0775	0.1925	0.1925	0.1925
Objective Detection	20	20	20	20	20	20
NA Objective detection NA	0.5	0.5	0.5	0.5	0.5	0.5
Field Of View (µm)	245	245	251	480	480	480
Light Sheet Thickness (µm)	4	4	4.8	1.4	1.4	1.9
Imaged volume (xyz) (voxels)	532x532x400	532x532x400	532x532x400	532x532x400	532x532x400	532x532x400
Lateral pixel size (µm)	0.26	0.26	0.26	0.26	0.26	0.26
Axial section (step size, µm)	1	1	1	1	1	1
Number of planes	400	400	400	400	400	400
Excitation wavelenght	532	532	1040	532	532	1040
Laser Power (mW) (@backfocal plane)	1.2mW	0.65mW	300mW	0.65mW	1.63mW	450mW
Exposure Time (ms)	100	100	1000	100	1000*	2000
Time to acquire a stack (min)	1	1	10	1	10	15

* 3ms/line

Supplementary Table 4: Experimental imaging parameters for the second stage of experiments

Modality	**G1P**	**B1P Cls**	**B2P**
Objective Magnification	10x	10x	10x
NA Illumination	0.07	0.27	0.27
Objective Detection	20	20	20
NA Objective detection	0.5	0.5	0.5
Field Of View (µm)	200	236	200
Light Sheet Thickness (µm)	5	5	6
Imaged volume (xyz) (voxels)	532x532x400	532x532x400	532x532x400
Lateral pixel size (µm)	0.26	0.26	0.26
Axial section (step size, µm)	1	1	1
Number of planes	400	400	400
Excitation wavelength	532	532	1040
Laser Power (mW) (@backfocal plane)	5µW	50µm	500mW
Exposure Time (ms)	500	500 *	500

* 3ms/line

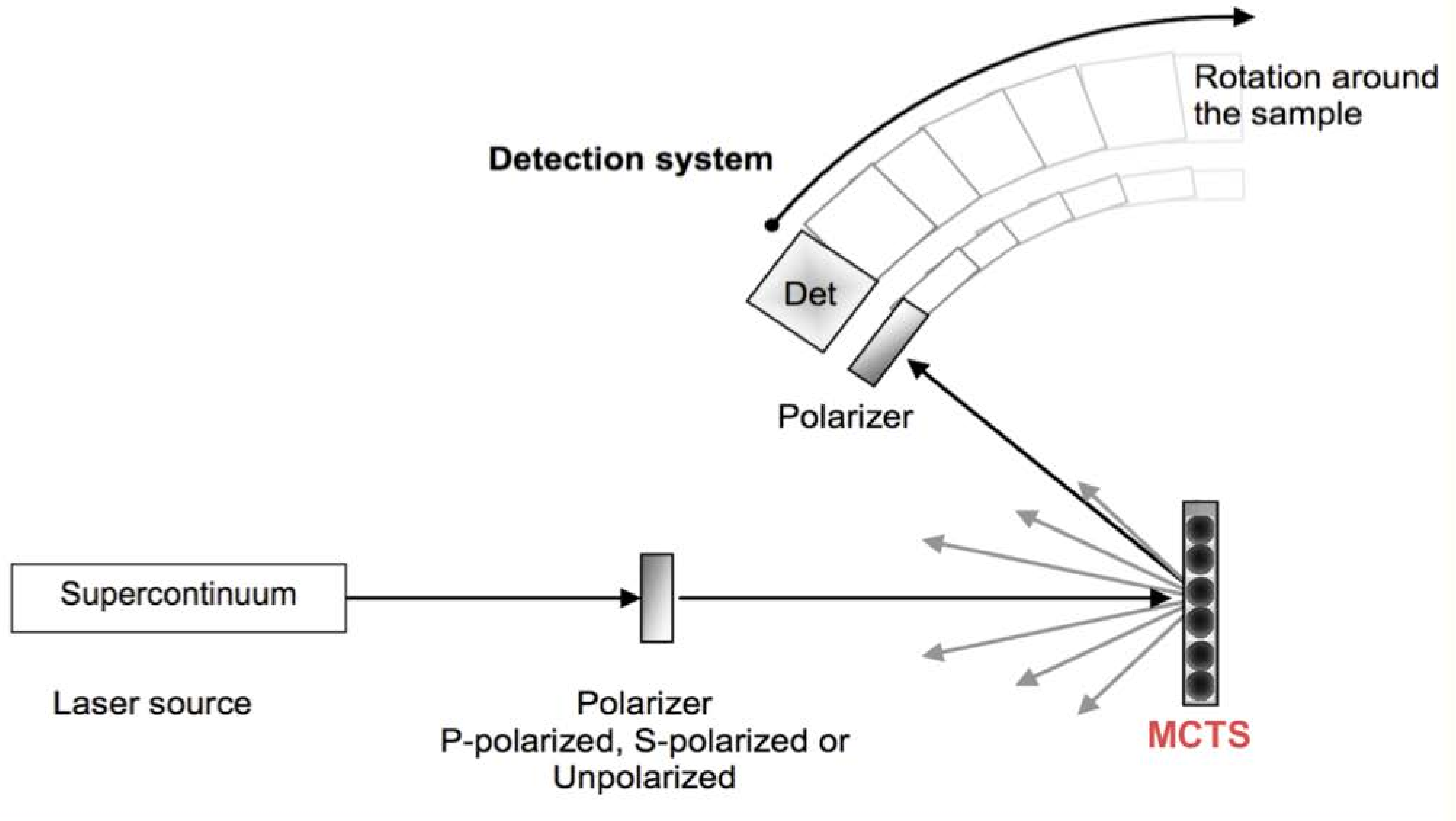

Supplementary Figure 1 : Schematic of the Melopee instrument adapted for TM measurements. The incident lighting system is a supercontinuum laser coupled to wide-band polarizers. The sensing system merges spectroscopy, polarimetry and scatterometry on a single goniometer platform.

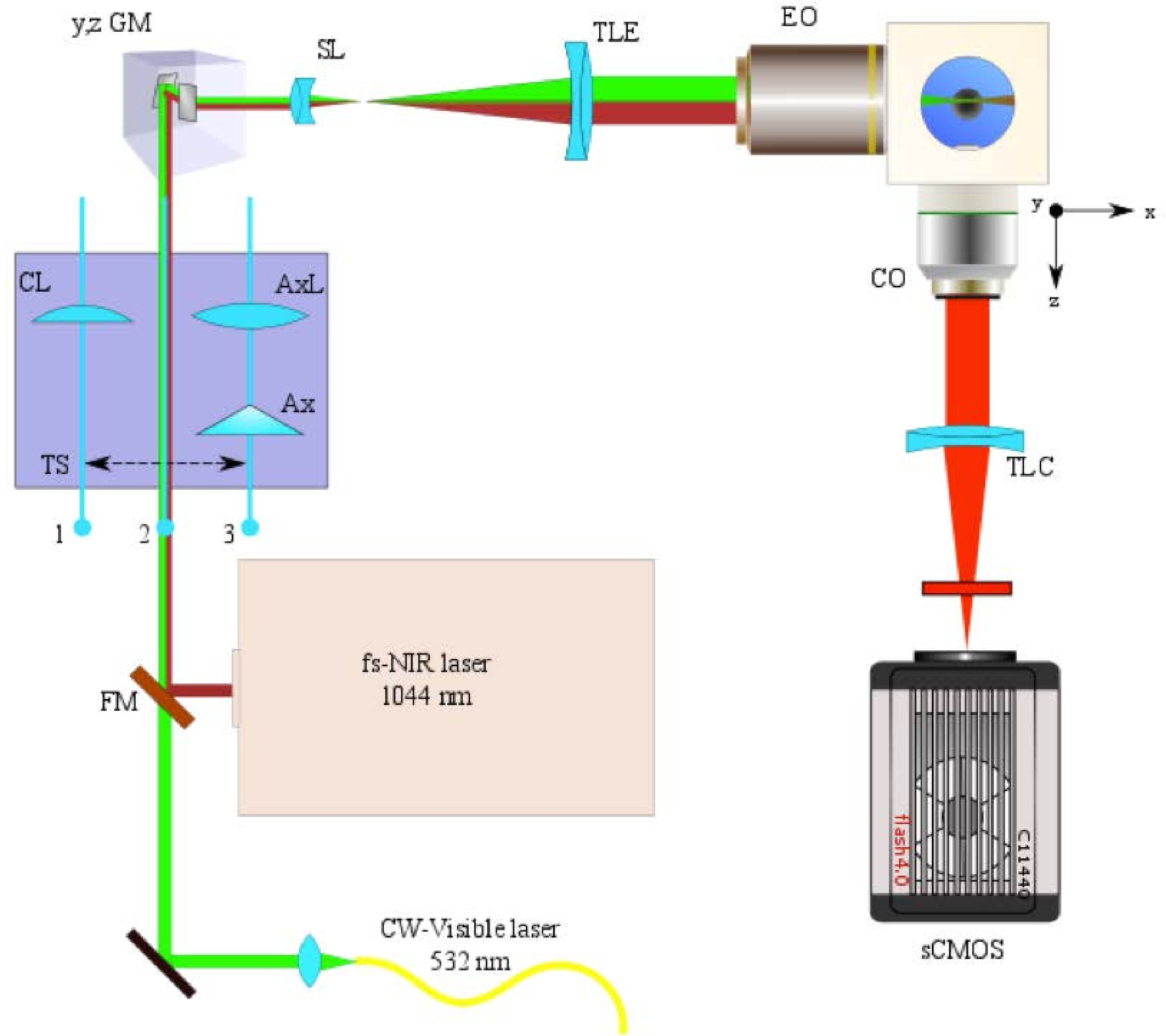

Supplementary Figure 2: Single Multimodal-LSFM setup as described in supplementary information 3. Main parts are listed in supplementary table 2.

Supplementary Figure 3: Beam profiles. Cross-sectional views (xy) of intensity profiles for different types of excitation beams propagating through agarose. Beams propagate from right to left in the images. (a,) correspond to the sketch of the geometry and the intensity profile views of beam at the center, of an agarose. Background is a white light image. ; (b-e) Profiles of the intensity signal of the longitudinal extent (y) with the point of maximum intensity located at the origin of coordinates corresponding to the cross- sectional view for different beams propagating through a 1 % agorose cylinder stained with Sulfo Rhodamine B.

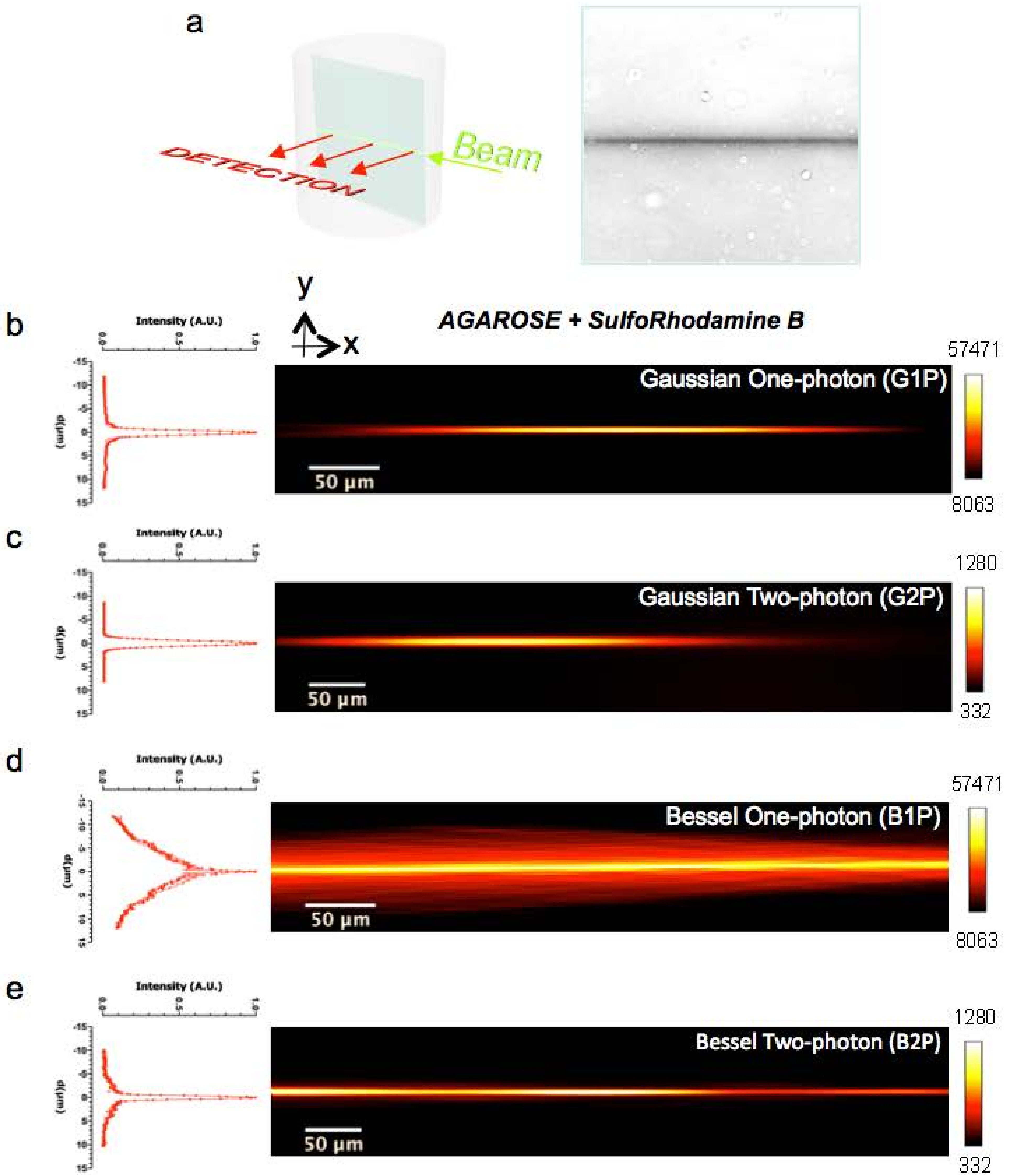

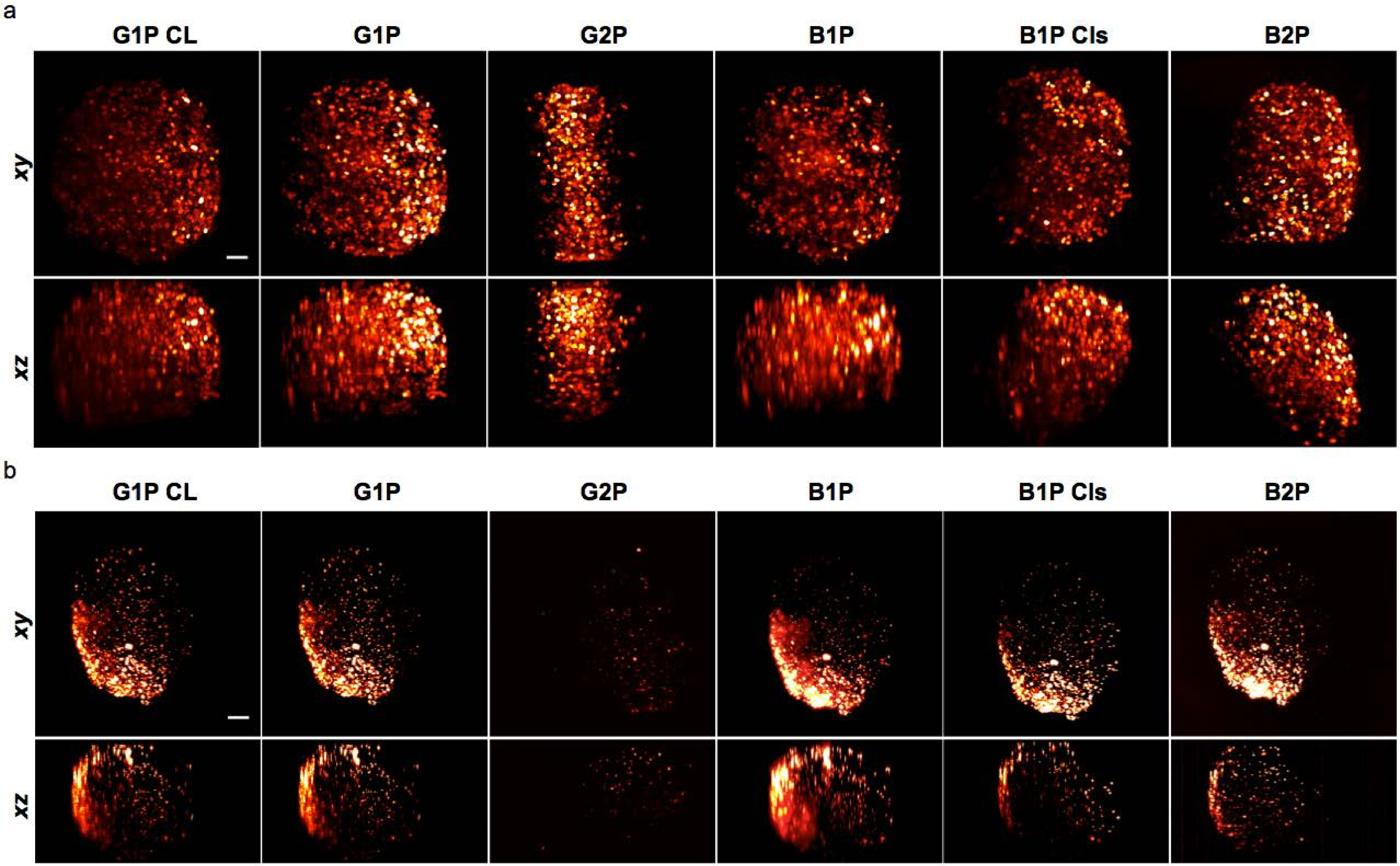

Supplementary Figure 4: **MCTS imaged with the six illumination modalities.** Maximum intensity projection along (x-y) and (x-z) of 3D image stack obtained using the indicated one- side light sheet illumination modality of (a) 400 images (z spacing 1μm) of MCTS measuring 400 μm in diameter and expressing a nuclear fluorescent protein (mCherry- H2B) and (b) of 300 images (z spacing 1μm) of MCTS measuring 300 μm and cultivated in presence of 0.5 μm fluorescent beads. For a good visualisation, scale intensity signals were set independently for each of modality. Scale Bar, 50 μm.

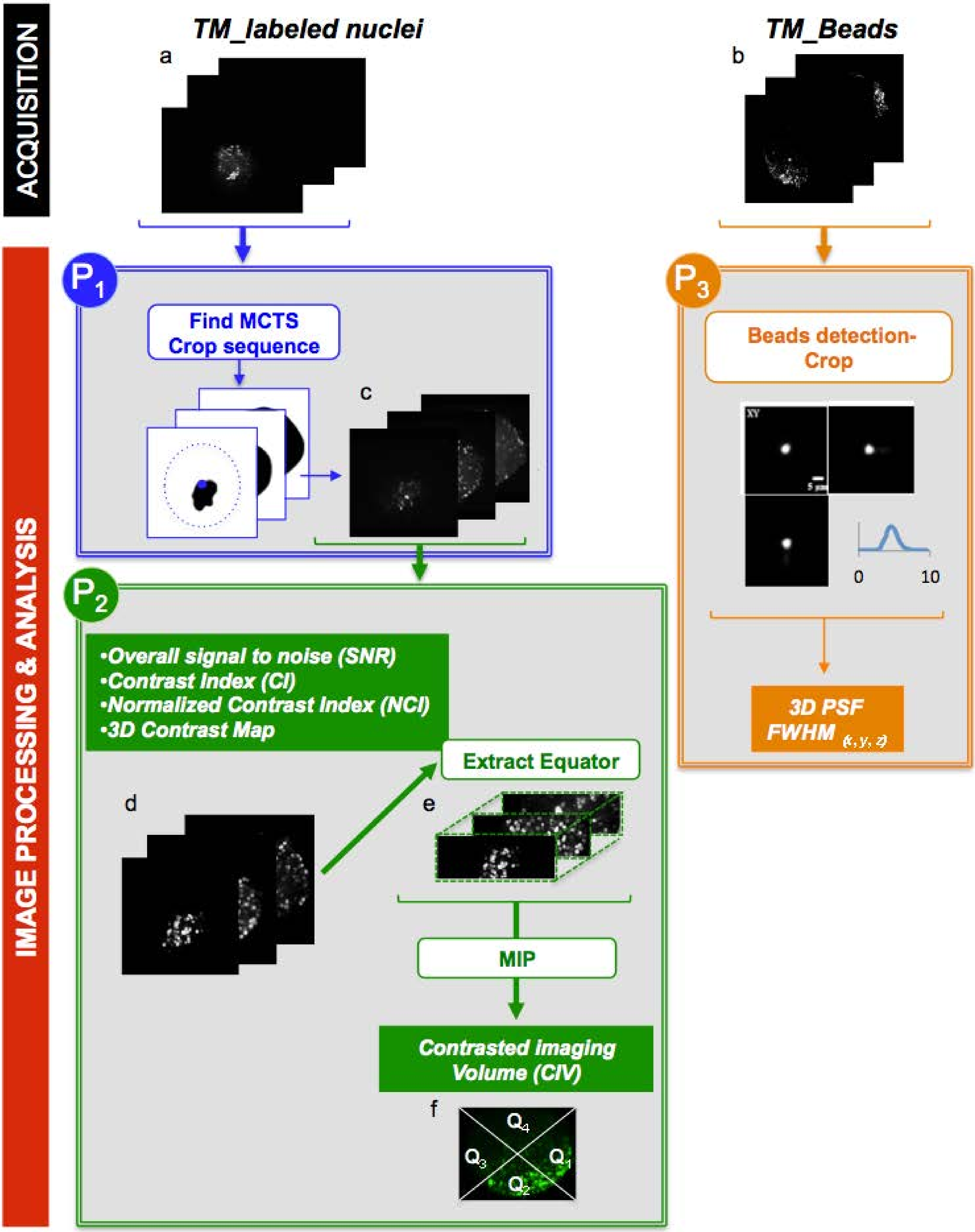

Supplementary Figure 5: Image Analysis framework

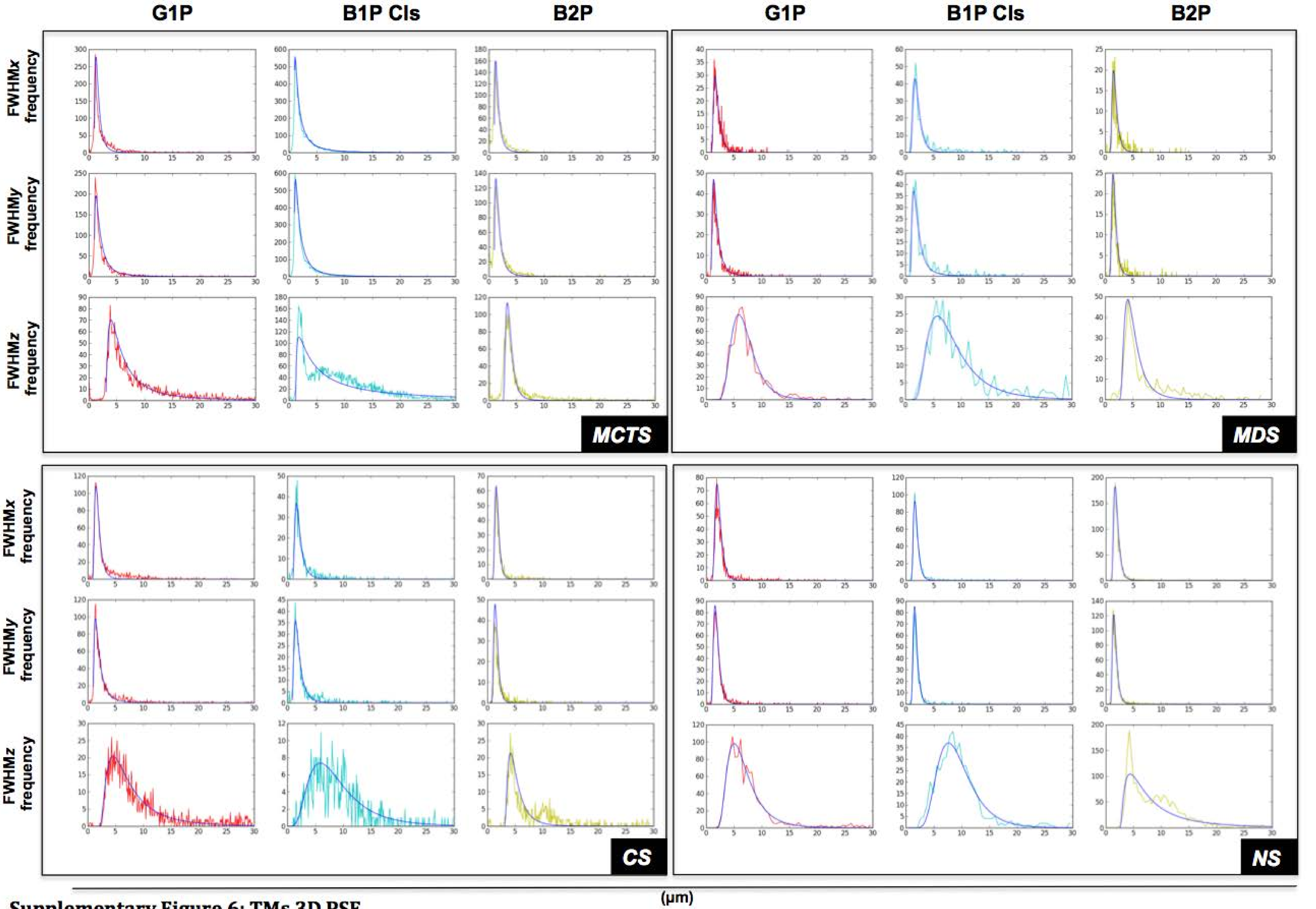

Supplementary Figure 6: TMs 3D PSF
